# Exploring Diverse Coagulation Factor XIII Subunit Expression Datasets: A Bioinformatic Analysis

**DOI:** 10.3390/ijms23094725

**Published:** 2022-04-25

**Authors:** Muhammad Ahmer Jamil, Sneha Singh, Osman El-Maarri, Johannes Oldenburg, Arijit Biswas

**Affiliations:** Institute of Experimental Hematology and Transfusion Medicine, University Hospital of Bonn, Building 043, Venusberg Campus 1, 53127 Bonn, Germany; muhammad.jamil@ukbonn.de (M.A.J.); sneha.gupta@ukbonn.de (S.S.); osman.elmaarri@ukb.uni-bonn.de (O.E.-M.); johannes.oldenburg@ukbonn.de (J.O.)

**Keywords:** FXIII, expression profiling, micro-array, RNA-Seq, differential gene expression, TRANSFAC, gene ontology, ingenuity pathway analysis

## Abstract

Coagulation factor XIII (FXIII) circulates in plasma as a pro-transglutaminase heterotetrameric complex (FXIIIA_2_B_2_), which upon activation by thrombin and calcium covalently crosslinks preformed fibrin polymers. The heterotetrameric complex is composed of a catalytic FXIIIA_2_ subunit and a protective/regulatory FXIII-B_2_ subunit coded by *F13A1* and *F13B* genes, respectively. The catalytic FXIIIA_2_ subunit is encoded by the *F13A1* gene, expressed primarily in cells of mesenchymal origin, whereas the FXIIIB subunit encoded by the *F13B* gene is expressed and secreted from hepatocytes. The plasma FXIIIA_2_ subunit, which earlier was believed to be secreted from cells of megakaryocytic lineage, is now understood to result primarily from resident macrophages. The regulation of the FXIII subunits at the genetic level is still poorly understood. The current study adopts a purely bioinformatic approach to analyze the temporal, time-specific expression array-data corresponding to both the subunits in specific cell lineages, with respect to the gene promoters. We analyze the differentially expressed genes correlated with *F13A1* and *F13B* expression levels in an array of cell types, utilizing publicly available microarray data. We attempt to understand the regulatory mechanism underlying the variable expression of FXIIIA_2_ subunit in macrophages (M0, M1, M2 and aortic resident macrophages). Similarly, the FXIIIB_2_ subunit expression data from adult, fetal hepatocytes and embryonic stem cells derived hepatoblasts (hESC-hepatoblast) was analyzed. The results suggest regulatory dependence between the two FXIII subunits at the transcript level. Our analysis also predicts the involvement of the FXIIIA_2_ subunit in macrophage polarization, plaque stability, and inflammation.

## 1. Introduction

To prevent hemorrhagic trauma upon injury, activated fibrin molecules assemble to form strong mesh-like structures known as fibrin clot. Although the generation of fibrin clot marks the end of the blood coagulation cascade, the resulting soluble clot is still susceptible to premature fibrinolysis. The conversion of this soluble clot to an insoluble one resistant to premature fibrinolysis is ensured by the crosslinking of pre-formed fibrin polymers by coagulation Factor XIII (FXIII), by virtue of its transglutaminase activity. In plasma, FXIII exists with the heterotetramer zymogenic complex with dimeric catalytic FXIIIA subunits, and dimeric protective FXIIIB subunits bind non-covalently to each other. In plasma, the overall abundance of this pro-transglutaminase complex (FXIIIA_2_B_2_) is ≈68 nM, with the two subunits bound with high affinity (Kd 1.5 nM) [1]. Upon activation by thrombin and sequential calcium-binding, this complex dissociates, releasing the catalytically active monomeric FXIIIA subunit for cross-linking fibrin [2]. Only an insignificant amount of free FXIIIA is present in plasma, although there are almost two times the amount of FXIIIB in plasma compared to FXIIIA subunit, thereby resulting in a significant amount of free the FXIIIB subunit. Plasma FXIIIA is now understood to be contributed to by cells of myeloid lineage derived from the bone marrow, although it is expressed in several other cell types such as monocytes, monocyte-derived macrophages, and platelets. The FXIIIB subunit is expressed in hepatocytes [3]. The zymogenic FXIII circulates in plasma bound to fibrinogen (Kd 10 nM) [4]. Other than covalently crosslinking fibrin molecules to itself, FXIII crosslinks other fibrin stabilizers such as (α 2-PI, PAI, TAFI, etc.) to the growing fibrin clot as well [5,6]. The deficiency of FXIII, inherited or acquired, is known to cause bleeding predispositions, where congenital FXIII deficiency is due to defects either of the *F13A1* (at genetic locus 6p24-25) or *F13B* genes (at genetic locus 1q32-32.1). The global prevalence of inherited FXIII deficiency is low (1–4 cases per million), which brings it under the category of rare bleeding disorders. Most defects associated with severe bleeding symptoms in inherited FXIII deficiency are largely due to defects in the *F13A1* gene, whereas gene defects in the *F13B* gene have been shown to be causing mild to moderate symptoms [7]. More than 100 distinct FXIII mutations from *F13A1* and *F13B* genes have been identified in patients with a broad spectrum of pathological phenotype severity that includes post-operative prolonged bleeding, delayed re-bleeding, and spontaneous abortion during the first trimester of pregnancy due to placental dysfunction. Over 500 cases of severe FXIII deficiency have been reported worldwide. In the past decade, clinical data have shown that although inherited FXIII deficiency is autosomal recessively inherited, the carriers or the heterozygous state of some of these FXIII gene defects can also bleed unusually when exposed to physical trauma [8].

Apart from its role in coagulation, several other roles outside coagulation have been discovered for the catalytic FXIIIA subunit. These roles encompass wound healing, chronic inflammatory bowel diseases, atherosclerosis, rheumatoid arthritis, chronic inflammatory lung diseases, chronic rhinosinusitis, solid tumors, hematological malignancies, and obesity [9]. Our group has previously reported on the novel potential binding partners of FXIIIB subunit based on co-immunoprecipitation studies i.e., α2-MG and complement factor C1q [10]. However, we could not find the direct roles of FXIIIB in complement activation in spite of its strong sequence homology and expected structural similarity to the complement factor H protein [10,11]. The FXIIIA subunit is expressed in a wide range of cells, including platelets, megakaryocytes, monocytes, and monocyte-derived cells, while the primary source of the FXIIIB subunit is hepatocytes [3,12]. Recent reports on FXIIIA expression and secretion exclude platelets and all their precursors as a major source of plasma FXIIIA. Although intracellularly the level of expression, activation, and secretion of FXIIIA is independent of its plasma partner FXIIIB subunits, it has been reported in several cases that the defect/reduction in one subunit often is accompanied by a reduction in levels for the other subunit as well. While this effect is anticipated for *F13B* mutations, because the FXIIIB subunit is the protective partner in the heterotetrameric complex, it is a little bit harder to explain the same observation for defects in the *F13A1* gene. This compels one to investigate if there is any co-regulatory mechanism shared by the two subunits that are expressed in different lineages yet define the final available dosage of potentially active FXIII transglutaminase in plasma. Previously, it has been reported that the pro-inflammatory M1 macrophages show lower to no expression of *F13A1*, compared to anti-inflammatory M2 macrophages [13]. In this context, *F13A1* has also been reported to be associated with carcinomas lately [14]. In several reports published earlier, based on the RNA-seq data, groups have reported a correlation of *F13A1* in diabetes-prone mice, osteoarthritis, and cancer, as one of the significant hits affected by altered cytokine signaling in these pathological states, indicating its role in these inflammatory states. A study performed in 2005 reported *F13A1* differential gene expression during macrophage polarization by RT-PCR and single-cell analyses [15]. The revelation that FXIIIA protein could be used as an intracellular marker for alternatively activated macrophages (the fully polarized classically (type I) and alternatively (type II) activated macrophages) compels one to look deeper at whether *F13A1* has any direct role in macrophage maturation and polarization. 

The current availability of abundant data being submitted in expression databases such as gene expression omnibus (GEO) provides the opportunity to bioinformaticians to investigate trends and patterns within these datasets, which in turn provide us with valuable insights into several functional and physiological mechanisms. Advanced bioinformatics tools and increased computational power have made it possible for us to understand the dynamic behavior of these biological data sets. To understand the roles and involvement of FXIII subunits in dynamic biological processes intra and extracellularly considering the overall gene expression as read-outs, such as hepatocyte maturation, the development of atherosclerotic plaque, and macrophage polarization, we have utilized the publicly available RNA-microarray data sampled at different time points to understand and capture expression-switching with respect to time, and the behavior of transcriptome. Since the cells of bone-marrow and mesenchymal lineage have so far been reported to be responsible for stable *F13A1* expression, we have investigated the micro-array data derived from macrophages, which are also major players in immunity. Similarly, microarray data derived from hepatocytes is investigated for *F13B* expression behavior. Recent reports on reduction in plasma levels of FXIIIA, upon acquired deficiency of FXIIIB subunit, also motivates one to investigate the pathways responsible for the common regulation of the two subunits [16]. This study, however, explains that such an acquired response does not affect the FXIIIA pool within the platelets. Interesting reports on platelet FXIIIA characterization also suggest that platelet FXIIIA is not responsible for the maintenance of the plasma pool of FXIII. After the identification of differentially expressed genes (DEGs) correlating with *F13A* and *F13B* expression levels in respective datasets, a common regulatory pathway is traced by characterizing the promoters and transcriptional regulators at each time point and cell type. To address the mode of FXIII expression regulation, for both of its subunits here we attempted to adopt a pure bioinformatics approach to analyze the temporal, time-specific expression array data corresponding to both the subunits in specific cell lineages with respect to the gene promoters. Differentially expressed genes with respect to the expression of the FXIII subunit genes in different cell lineages were predicted for their potential roles in cellular pathways. 

## 2. Materials and Methods

### 2.1. Data Extraction

Expression data were extracted from NCBI GEO (GSE128303, GSE98324, GSE39157, and GSE41571) for macrophages (time-series), hepatic progenitor, hepatocytes, and human-embryonic-stem-cells-derived hepatoblasts, respectively, [13,17,18,19,20,21]. GSE128303 and GSE98324 were available for illumina human ht12-v4 expression beadchip arrays, whereas GSE39157 and GSE41571 were analyzed using Affymetrix Human Gene 1.0 ST arrays (HuGene-1_0-st) and Affymetrix Human Genome U133 Plus 2.0 Array (HG-U133_Plus_2). As a summary, we put together the following purified human-derived expression data sets:

***GSE128303:*** In the original study corresponding to this dataset, primary human monocyte-derived macrophages isolated from four donors were matured in the presence of recombinant human IL-4, followed by LPS treatment (*Salmonella enterica*, 200 ng/mL) for 90 min [17]. LPS-treated samples were diluted to a final concentration of 100 ng/mL LPS for the time following the initial 90-min incubation. Samples were collected at 6 h and 24 h post-initiation of infection for a total of 12 treatments in biological quadruplicate for 48 total samples. In this present analysis, out of the 12 treatments performed by Miller et al. resulting in 48 samples, we filtered 8 samples (LPS treated) derived from 4 donors, one for each time point (6 h and 24 h). *Platform: Illumina HumanHT-12 V4.0 expression beadchip*.

***GSE98324:*** In the original study corresponding to this dataset, research groups at the National University of Singapore collected array data from 32 distinct human-derived samples [18]. From this comprehensive data set, we have used the array data for four samples attributed to hESCs (Human ESC-derived Hepatic Progenitor Under Base Condition). *Platform: Illumina HumanHT-12 V4.0 expression beadchip*.

***GSE39157:*** In the original study corresponding to this dataset, array data derived from total RNA were obtained from cultured primary hepatocytes and hESC-derived hepatic populations [19]. In the present study, we have used the primary hepatocyte and hESC-derived untreated hepatoblast culture, derived expression array data. *Platform: (HuGene-1_0-st) Affymetrix Human Gene 1.0 ST Array (transcript (gene) version).*

***GSE41571:*** In the original study corresponding to this dataset, expression data derived from genome-wide expression analyses of isolated macrophage-rich regions of stable and ruptured human atherosclerotic plaques were reported [20,21]. *Platform: (HG-U133_Plus_2) Affymetrix Human Genome U133 Plus 2.0 Array.*

### 2.2. Data Processing

***Illumina Human HT12-v4 Expression BeadChip*:** Raw data from GEO was downloaded as “idat” files for illumina human ht12-v4 platform. IDAT files were imported into R using “read.idat” function in “Iimma” package [22]. Data were background-corrected and quantile-normalized using “neqc” function in “limma” package. Finally, data were filtered for probes not detected in any cell type by using “detectionPvalues” method; this method identified the observed expression values of probes in comparison to the negative control probes and provides us with the *p*-values for probe expression. Data were also tested for batch effects using “ComBat” function in “sva” package, but no batch effect was removed as the data has a non-significant batch effect [23].

***Affymetrix Arrays****:* The normalized series matrix file was downloaded from the NCBI GEO database for both Affymetrix array data, and this file was filtered for the detection *p*-value, where the probe signal was found to be statistically significant against background signal. Probes with non-significant probe signals were removed, and significant probes were further analyzed using the Qlucore Omics Explorer 3.5 (www.qlucore.com (accessed on 6 Jun 2020)) (Qlucore AB, Lund, Sweden).

***Data Import***: All expression arrays data were imported separately into Qlucore Omics Explorer 3.5 (www.qlucore.com (accessed on 6 Jun 2020)) using import wizard with sample annotation, as well as probes annotation for individual array platforms.

### 2.3. Differential Gene Expression Analysis

Differentially expressed genes (DEG) were identified using Qlucore Omics Explorer 3.5 (www.qlucore.com (accessed on 6 Jun 2020)). A Student t-test was used to compare two samples, whereas an ANOVA was used for multiple-sample comparison. Significance values of *p* < 0.05 or 5% of the false discovery rate were used as statistically significant. Fold changes were also calculated for volcano plots.

***Gene Ontology and Pathway Analysis:*** Gene ontology analyses were carried out using Cytoscape 3.7.1 [24]. Biological processes were identified using “Bingo” plugin in Cytoscape [25]. Visualizations of significant ontologies were carried out using the “EnrichmentMap” plugin in Cytoscape [26]. Tox functions were identified using ingenuity pathway analysis (IPA) (QIAGEN Inc., Cambridge, MA, USA, https://www.qiagen-bioinformatics.com/products/ingenuity-pathway-analysis (accessed on 6 Jun 2020)) [27].

***Promoter Sequence:*** The *F13A1* and *F13B* promoter sequences were downloaded from the eukaryotic promoter database (EPD, www.epd.epfl.ch (accessed on 6 Jun 2020)) [28,29]. The promoter sequence of 1 kb upstream and 1 kb downstream from the transcription start site was downloaded from EPD, and a 2 kb long sequence was downloaded as a “fasta” file for further transcription factor analysis.

***Transcription factors analysis:*** The transcription factors binding to the promoter of *F13A1* and *F13B* downloaded from EPD were identified using the TRANSFAC professional database from geneXplain [30,31,32]. Transcription factor binding sites were identified as transcription factor matrices (TF-matrices; each transcription factor matrix is comprised of multiple transcription factors with same binding sites; similarly, one transcription factor could bind to multiple binding sites. All TRANSFAC analyses were carried out using the data version of 2019.3 with the selected profile of matrices with a minimum sum of false positive and false negative, and only high-quality matrices were used for the analyses [32]. Further analyses of transcription factors matrices were carried out using R.

### 2.4. Visualization and Plots

Principal component analysis and heatmaps were generated using Qlucore Omics Explorer 3.5 (www.qlucore.com (accessed on 6 Jun 2020)). Volcano plots and correlation plots were generated using R programming. Box plots were generated using the GraphPad Prism 8. Pathways, gene networks, and tox functions plots were created using Ingenuity Pathway Analysis. Gene ontology plots were generated using Cytoscape 3.6. Transcription factors binding consensus sequences were downloaded from TRANSFAC.

## 3. Results

### 3.1. Genes Correlated with F13A1 and F13B Have Specific Biological Processes as well as a Common Function of Immune Response

**Figure 1:**
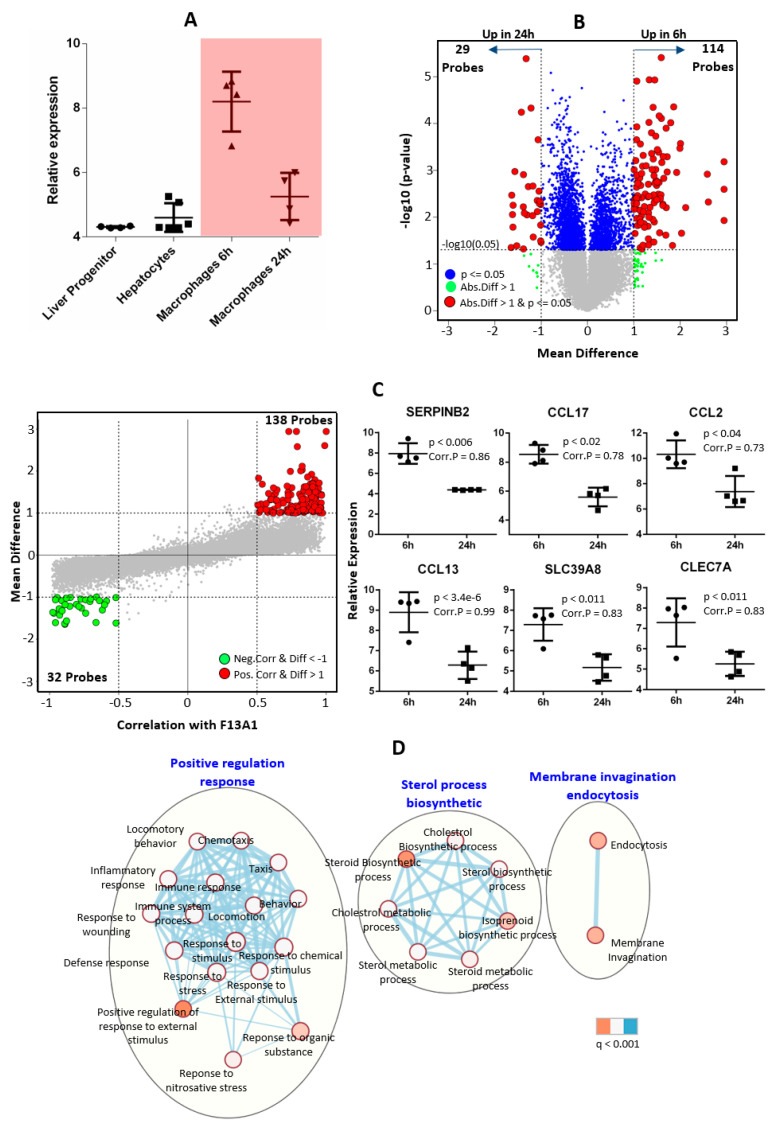
An expression analysis of time-series macrophages (LPS treated) at 6 h and 24 h. (**A**) A box plot of *F13A1* expression in macrophages at 6 h and macrophages at 24 h, as well as liver progenitor cells and hepatocytes. (**B**) A volcano plot of differentially expressed genes between 6 h and 24 h macrophages (red: significant *p*-value < 0.05 and absolute mean difference above 1; green: absolute mean difference above 1 and *p*-value > 0.05; and blue: significant *p*-value < 0.05 at absolute mean difference < 1) in dataset *GSE128303*. (**C**) Left: Bland–Altman plot between the correlation of *F13A1* expression with other genes and the mean difference expression of genes between macrophages 6 h and 24 h; red = significantly positively correlated genes at Pearson’s correlation > 0.5 and absolute mean difference between 6 h and 24 h > 1; and green = significantly negatively correlated genes at Pearson’s correlation < −0.5 and absolute mean difference between 6 h and 24 h > 1. Right: top 6 markers from the Bland–Altmann plot between 6 h and 24 h. (**D**) Gene ontology (GO) analysis of significant genes from the Bland–Altman plot in part **C**-Left.

**Figure 2:**
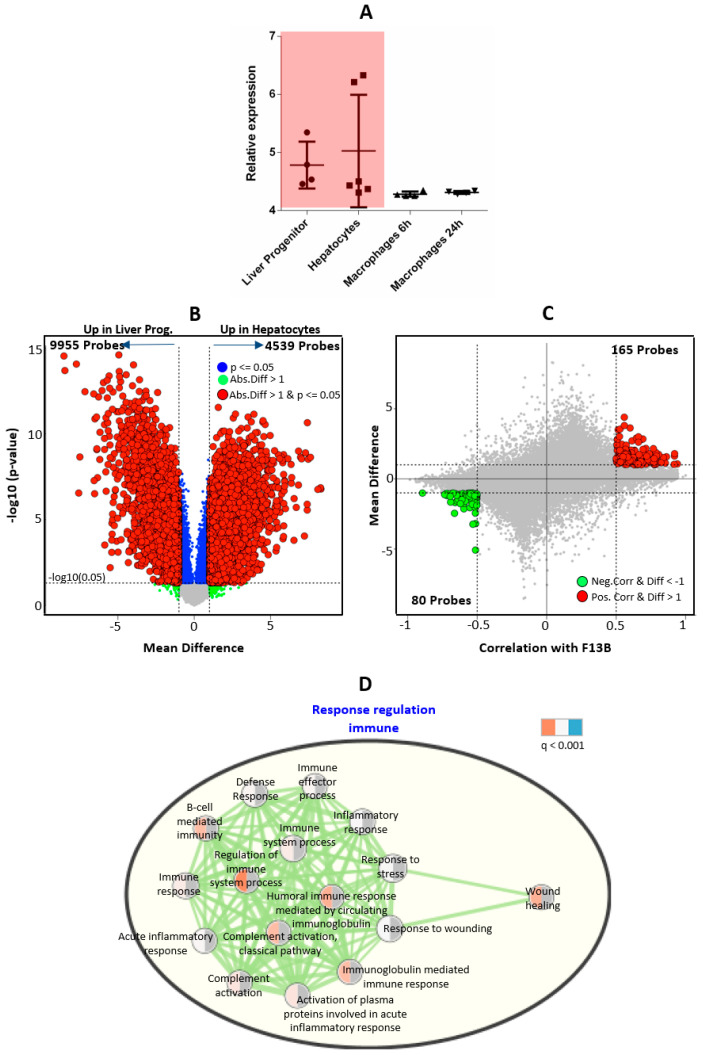
An expression analysis of *F13B* in liver progenitor cells and hepatocytes. (**A**) A box plot of *F13B* expression in liver progenitor cells, hepatocytes, and macrophages. (**B**) A volcano plot of differentially expressed genes between liver progenitor cells and hepatocytes at red: significant *p*-value < 0.05 and absolute mean difference between liver progenitor and hepatocytes above 1; green: the absolute mean difference between liver progenitor and hepatocytes above 1 but *p*-value > 0.05; and blue: statistical *p*-value < 0.05, but the absolute mean difference between liver progenitor and hepatocytes < 1. (**C**) The Bland–Altman plot between the correlation of *F13B* expression with other gene expressions and the mean expression difference of the genes between liver progenitor cells and hepatocytes; red: significantly positively correlated genes at Pearson’s-Correlation > 0.5 and the absolute mean difference between liver progenitor and hepatocytes > 1; green: significantly negatively correlated genes at Pearson’s Correlation < −0.5 and the absolute mean difference between liver progenitor and hepatocytes > 1. (**D**) A gene ontology analysis of significant genes from the Bland–Altman plot in part (**C**).

The *F13A1* and *F13B* genes were observed to be expressed in macrophages and liver cells, respectively. Reversibly, no expression for the *F13A1* and *F13B* genes was observed in macrophages and liver cells, respectively. The FXIIIA subunit expression reduces significantly with the maturation of macrophages. Macrophages at 6 h (monocyte-derived macrophages) showed high levels of the *F13A1* transcript, which after 24 h goes down to ≈50% of its original expression (Figure 1A). Overall, macrophages showed 143 probes (114 probes upregulated at 6 h, 29 probes upregulated in 24 h) to be differentially expressed across 6 h and 24 h time-points at *p* ≤ 0.05, with a mean difference greater than 1. Additionally, 170 probes (positive correlated = 138 probes, negative correlated = 32 probes) were found to be significantly correlated with *F13A1* expression in macrophages at correlation > 0.5 and with a mean difference greater than 1 (Figure 1B). The top hits reveal genes such as *SERPINB2, CCL17, CCL2, CCL13,* and *SLC39A8* having a strong positive correlation to *F13A1* expression (>0.5 Pearson’s correlation) (Figure 1C). The gene ontology analysis of negatively correlated genes showed no significant biological process, whereas positively correlated genes showed strong enrichment of response-based terms (i.e., immune response, stress response, response to wounding, etc.) (a detailed ontology list is shown in Figure 1D), sterol process biosynthetic, and endocytosis. *F13B* expression is not significantly different between liver progenitor and hepatocytes, but mildly high expression was observed in adult hepatocytes. No *F13B* transcript was observed in macrophages (i.e., above detection level) (Figure 2A). Liver progenitor and hepatocytes are very different cell types since liver progenitor cells differentiate into diverse cell types whereas hepatocytes are liver-specific cells. Differential gene expression analysis between hepatocytes and liver progenitor cells showed a high number of differentially expressed genes (14494 probes at *p* < 0.05 and mean difference greater than 1) (Figure 2B). Meanwhile, FXIIIB expression was showing non-significant expression difference between hepatocytes and liver progenitor; hence, only 245 probes (positive correlated = 165 probes, negative correlated = 80) were found to be significantly correlated (correlation > 0.5) with FXIIIB expression and have a mean difference above 1 between liver progenitor and hepatocytes (Figure 2C). The gene ontology analysis of correlated genes with *F13B* showed enrichment in terms of the immune response and wound healing-related biological processes (Figure 2D). The basal level of expression of *F13B* (in hepatocytes) as compared to *F13A1* (in macrophages) was ≈four times higher. This might explain the higher levels of the FXIIIB_2_ subunit in plasma compared to the FXIIIA_2_ subunit (≈2 times), owing to which there is a significant amount of free unbound FXIIIB_2_ subunit in the plasma.

#### 3.1.1. Genes for Both the FXIII Subunits Have a Common Inhibitor/Suppressor Transcription Factor Binding Site

**Figure 3:**
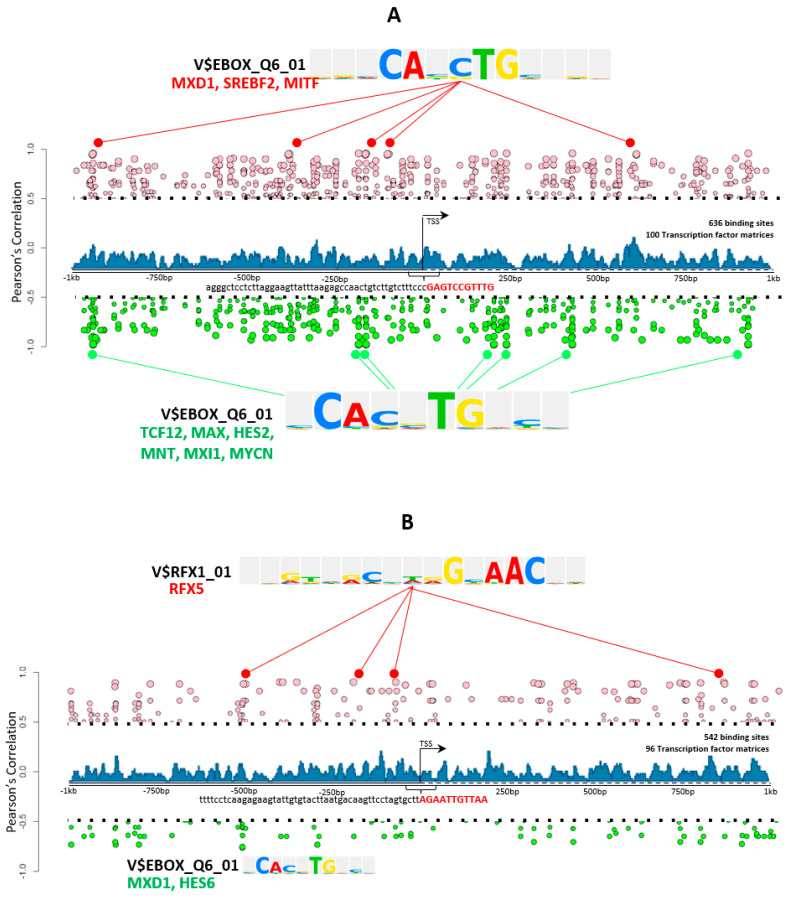
The transcription factor analysis of *F13A1* and *F13B* using TRANSFAC. (**A**) An illustration of the predicted transcription factor binding to the *F13A1* promoter; pink: the positively correlated expression of transcription factor with *F13A1* expression; green: the negatively correlated expression of transcription factor with *F13A1* expression. Blue: the density of the transcription factor at a particular position on the gene-promoter. A heatmap of transcription factors binding to a specific binding site represented as a transcription factor matrix. (**B**) An illustration of predicted transcription factor binding to the *F13B* promoter; pink: the positively correlated expression of the transcription factor with *F13B* expression; green: the negatively correlated expression of the transcription factor with *F13B* expression. Blue: the density of the transcription factor at a particular position. The above heatmap of the correlation between transcription factors and *F13B* expression; the below heatmap of transcription factors binding to a specific binding site represented as a transcription factor matrix.

**Figure 4:**
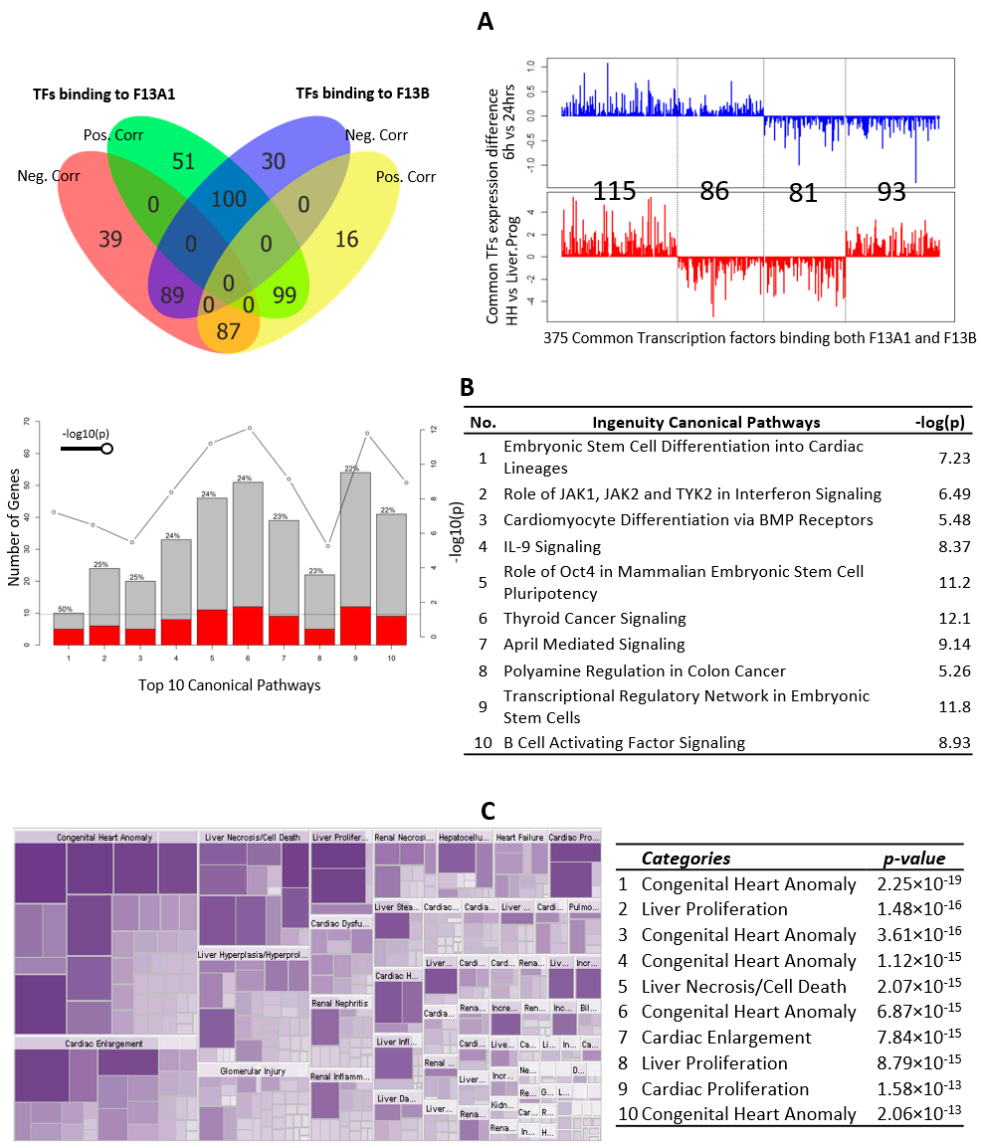
The common transcription regulation of F13A1 and F13B. (**A**) Left: the Venn Diagram of the positive and negative correlation of TFs binding to F13A1 and F13B, in macrophages 6 h vs. 24 h and liver progenitors cells vs. hepatocytes respectively. Right: the transcription factors expression difference in macrophage 6 h vs. 24 h and liver progenitor vs. hepatocytes for common transcription factors binding to both F13A1 and F13B. (**B**) The top 10 canonical pathways of common transcription factors binding to both F13A1 and F13B; red: the overlap transcription factor from our analysis; and grey: the total number of molecules in the pathway. (**C**) Left: the heatmap of all tox functions enriched with common transcription factors binding to both F13A1 and F13B; right: the Top 10 Tox functions of the common transcription factor binding to both F13A1 and F13B.

On analyses of the extended 2 kb long promoter for both *F13A1* and *F13B* genes, the transcription factors (TFs) binding to these extended promoters using TRANSFAC professionals, were identified. TRANSFAC predicted 100 and 96 TF-Matrices binding to 636 and 542 binding sites on *F13A1* and *F13B* promoters, respectively (see Supplementary Figure S1 and Supplementary Table S1). These matrices correspond to 465 and 421 probes in the expression data of macrophages and liver cells, respectively. Differential gene expression analysis of these transcription factors gave us 68 probes when macrophages at the 6 h time point were compared with macrophages at the 24 h time point. A comparison between liver progenitor and hepatocytes gave 202 probes of differentially expressed transcription factors (see Supplementary). Further, the correlation between the differentially expressed transcription factors of *F13A1* and *F13B* was calculated. It was found that the V$EBOX_Q6_01 transcription factor matrix was found both positively and negatively correlated with *F13A1* expression. In the given matrix “MXD1, SERBF2, and MITF” were found to be highly positively correlated with *F13A1* expression, whereas “TCF12, MAX, HES2, MNT, MXI1, and MYCN” were found to be highly negatively correlated (Figure 3A). Similarly, V$EBOX_Q6_01 also showed a statistically significant correlation with *F13B*, where “HES6” was found to be highly negatively correlated with *F13B.* The transcription factor binding elements “MXD1 and HES6” were found to be negatively correlated (with *F13B*) with a Pearson’s correlation below 0.75 and *p*-value < 0.01 (Figure 3B). A positive correlation was observed for V$RFX_01 binding sites, where the “RFX5” transcription factor was found to be highly positively correlated with *F13B* expression (Figure 3B). A closer comparison of these transcription factor binding elements revealed 375 TFs common to both *F13A1* and *F13B* genes (Figure 4A). A similar expression pattern was observed for 196 TFs (115 upregulated, and 85 downregulated) out of 375 common TFs in time series macrophages, as well as liver progenitor vs. hepatocytes, whereas the opposite pattern was observed for the remaining common 179 TFs (86 upregulated in time series macrophages, and 93 upregulated in hepatocytes vs. liver progenitor), which were predicted to bind to both *F13A1* and *F13B* yet show a reverse pattern of expression. An assessment of canonical pathways associated with all these ‘commonly regulating’ transcription factors revealed that the TFs correlated with F13 genes expression in the given cells are majorly responsible for cellular maturation, pluripotency, and development (Figure 4B). 

#### 3.1.2. Common TFs Binding to Both Subunits Are Related to Cardiac Anomaly

We have found that 80 transcription factor matrices were found to bind both *F13A1* and *F13B*. These 80 transcription factor matrices corresponded to 375 transcription factors. Tox functions from the ingenuity pathway analysis (IPA) of these 375 transcription factors showed enrichment in congenital heart anomaly, cardiac proliferation, cardiac enlargement, liver necrosis, and liver proliferation. This tox function shows that transcription factors binding to both *F13A1* and *F13B* play a significant role in pathological conditions such as cardiac anomaly (Figure 4C).

#### 3.1.3. F13A1 Expression Profiles Reveal Its Participation in Macrophage Polarization

**Figure 5:**
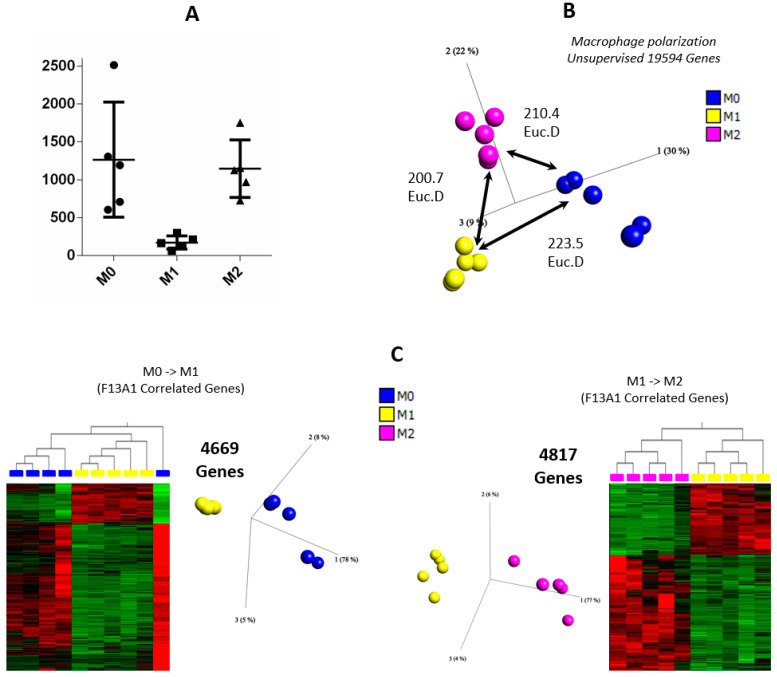

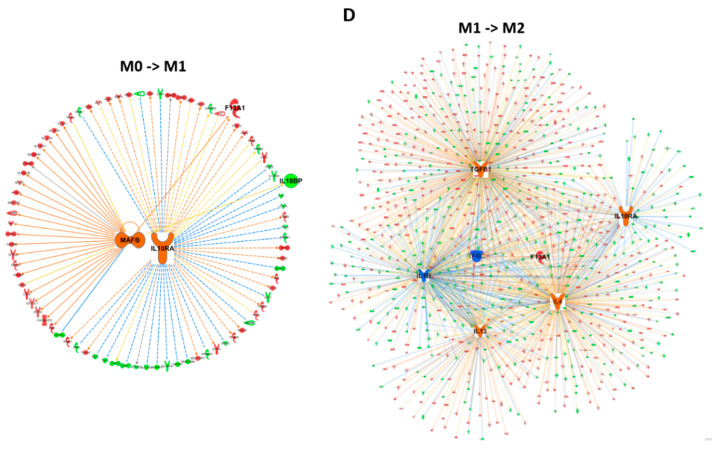
An expression analysis of macrophage polarization. (**A**) F13A1 expression in macrophage polarization. (**B**) The 3D-PCA of macrophage polarization with Euclidean distance (ED) between every macrophage polarization. Blue: M0; yellow: M1; and dark pink: M2. (**C**) A heatmap and 3D-PCA of significant F13A1-expression-correlated genes at *p* < 0.05; left: significantly correlated genes with F13A1 expression in M0 to M1; right: significantly correlated genes with F13A1 expression in M1 to M2. (**D**) Significant upstream regulators of F13A1-expression-correlated genes in left: M0 to M1; right: M1 to M2; orange: predicted overexpressed; blue: predicted under expressed; red: overexpressed; and green: under expressed in M0 compared to M1 or M1 compared to M2.

Relative gene expression of *F13A1* was significantly higher in M0 and M2 macrophages when compared to M1 (Figure 5A). A correlation gives us a measure of variability between two features with respect to the target variable, here being F13A expression. We found 4669 genes correlated to *F13A1* expression during the M0-M1 switch, whereas we found 4817 genes in the case of M1-M2 switching (Figure 5C). The upstream regulator analysis of *F13A1* and its correlated genes in both cases resulted in the prediction of common regulators (activation z scores >2 is considered significant). In the case of the M0-M1 switch, MAFB (z-score 3.945) and IL10RA (z-score 4.666) regulators are predicted to be inhibited in the genes that are correlated to *F13A1* expression, whereas in the case of the M1-M2 switch, IL1B (z-score 4.249) and TFRG (z-score 3.097) are the predicted regulators to be activated, and TGFB1 (z-score 3.902), IL10RA z-score 4.972), IL-13 (z-score 4.446), and IL-4 (z-score 4.078) are the regulators predicted to be inactivated in the genes correlated to *F13A1* expression (Figure 5D). 

#### 3.1.4. Stabilized Levels of F13A1 in Resident Macrophages Isolated from Ruptured and Stable Human Atheromatous Lesions

**Figure 6:**
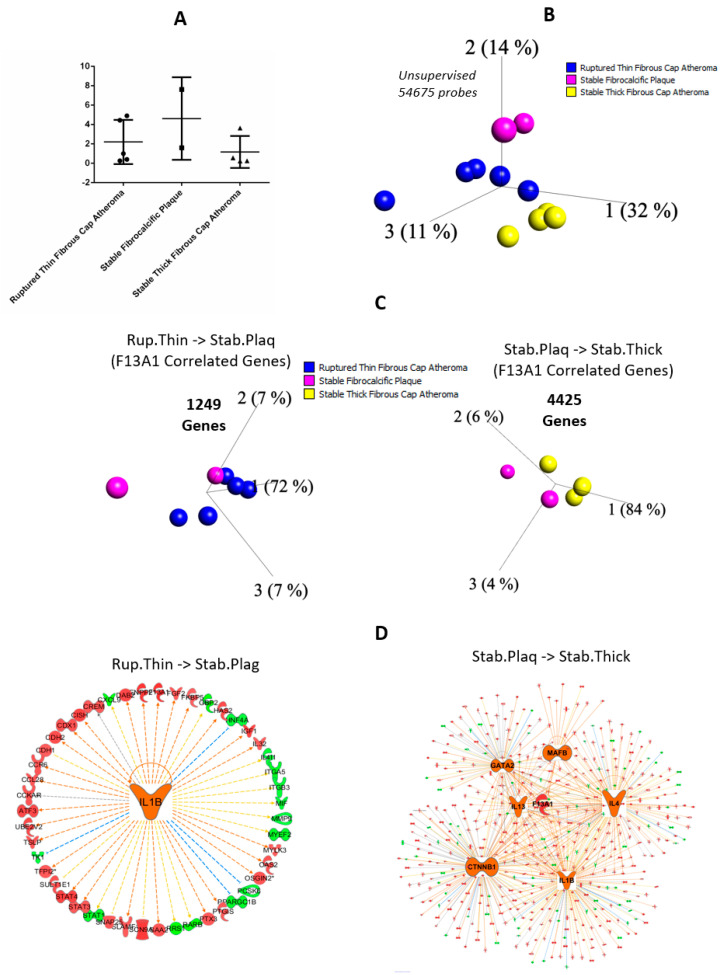
The expression analysis of plaque stability. (**A**) F13A1 expression in plaque stability. (**B**) 3D-PCA of plaque-stability-derived data points. Blue: Ruptured Thin Fibrous Cap Atheroma; yellow: Stable Thick Fibrous Cap Atheroma; and dark pink: Stable Fibrocalcific Plaque. (**C**) 3D-PCA of significant F13A1-expression-correlated genes at *p* < 0.05; left: significantly correlated genes with F13A1 expression in Ruptured Thin Fibrous Cap Atheroma to Stable Fibrocalcific Plaque; and right: significantly correlated genes with F13A1 expression in Stable Fibrocalcific Plaque to Stable Thick Fibrous Cap Atheroma. (**D**) Significant upstream regulators of F13A1-expression-correlated genes in left: Ruptured Thin Fibrous Cap Atheroma to Stable Fibrocalcific Plaque; right: Stable Fibrocalcific Plaque to Stable Thick Fibrous Cap Atheroma; orange: predicted overexpressed; blue: predicted under-expressed; red: overexpressed; and green: under-expressed in Ruptured Thin Fibrous Cap Atheroma compared to Stable Fibrocalcific Plaque or Stable Fibrocalcific Plaque compared to Stable Thick Fibrous Cap Atheroma.

No differential expression for *F13A1* was observed among the array data of resident microphages isolated from ruptured and stable human atheromatous lesions, most likely because all profiles were characteristic of activated macrophages, as also indicated by hierarchical clustering of the data set (Figure 6A,B). We found 1249 genes correlated to *F13A1* gene expression between the ruptured thin fibrous cap arthromere and stable plaques, whereas 4425 genes correlated to *F13A1* expression as in stable calcific plaque and Stable Thick Fibrous Cap Atheroma (Figure 6C). The upstream regulator analyses of the correlated genes predicted 1L1B (z-score 2.284) as the major regulator (activation z scores > 2 is considered significant) for these correlated genes for ruptured thin fibrous cap arthromere and stable calcific plaques, whereas, in case of progression from stable calcific plaques to Stable Thick Fibrous Cap Atheroma, IL4 (z-score 4.052), IL1B (z-score 4.272), IL13 (z-score 3.527), GATA2 (z-score 2.299), CTNNB1 (z-score 3.911), and MAF8 (z-score 3.327) were found to be significantly regulating the genes correlating to the *F13A1* expression pattern (Figure 6D).

## 4. Discussion

The NCBI-GEO (gene expression and molecular abundance repository) serves as one of the largest publicly available repositories for raw as well as curated array-based expression data, deposited by the scientific fraternity for further analyses, interpretation, and validation. Such large datasets enable and promote meta-analyses where raw data generated and deposited from one working group can be accessed, analyzed, and validated using several other bioinformatics tools to derive a better understanding of biological data. In the present study, we have utilized the GSE data records for different array-data analyzed here, which defines sets of samples and how they are related (here, for example, macrophages (M1, M2, and M0), liver cells (hepatocytes and liver progenitor cells), and plaque-derived resident macrophages (from stable and ruptured plaques)). This work is an attempt to translate bioinformatics data into meaningful interpretive FXIII research that could direct future investigations into the cellular expression profile of FXIII. 

The multitude of data sets described in the methods section is referred to and extracted for this present overview of FXIII subunit roles and its respective subunit expression, promoter specifications, and inter-related mode of regulation. We have tried to include the expression data from all stages of respective subunit-expressing cells (macrophages and hepatocytes, for *F13A1* and *F13B,* respectively). FXIIIA has been long been understood to be a key molecule in the inflammation-coagulation-complement axis. The bench data from several meta-analyses suggest that *F13A1* expression is strongly correlated to inflammation-like cellular responses; the temporal array data analyzed for cell-specific expression also reveal that the genes correlated with the *F13A1* and *F13B* differential expression pattern are largely involved in a common function of the immune response. The presented data here reveal that *F13A1* expression levels in macrophages are strongly positively correlated to genes responsible for cell differentiation and migration, largely in an immune setup (CCL17, CCL2, and CCL13); genes responsible for indirect clot stabilization (SERPINB2); and intracellular transporters such as SLC39A8 (Figure 1). These genes have earlier also been reported to act in synergy and to be involved in the coagulation-complement axis, e.g., in an earlier report, *F13A1* has been shown to be correlated with SERPINB2, MAF, IGF1, MAFB, and IL10, although the primary take-home message for this regarded the effect of Activin A on macrophage polarization, as a result of M-CSF based activation [33]. The observation that reduced plasma levels of the FXIIIB domain secondarily reduces the plasma levels of the available active FXIIIA domain is also translated into the curated array data here, where we see the basal expression of *F13B* genes almost four-times higher than that of *F13A1* (Figure 3 and Figure 4). The strong dependency of the two subunit plasma titers, which result in secondary deficiency of FXIIIB in the absence of FXIIIA in plasma and vice versa, draws attention towards the possible common regulation of the two subunits [34,35]. We observe that the FXIII subunit genes have a common inhibitor/suppressor predicted transcription factor binding site supporting the possibility of inter-regulation at the transcript level (Figure 4). This means that the defective activation of transcription in one partner gene might lead to no activation of transcription in the other partner. More experimental validation data in this context are needed to build upon this prediction. We further find that the Common TFs binding to both subunits are related to cardiac anomaly. The association of transcriptional regulation of the *F13A1* gene with cardiac anomaly does not come as a surprise for us since FXIII has often been related to defects in the cardiovascular system [36]. 

High levels of intracellular FXIIIA (in monocyte-derived macrophages) are reported to perform functions related to cellular remodeling, crosslinking of the cytoskeleton, microtubule assembly, etc. (given that FXIIIA has several substrates within the cell) [37]. However, similar high levels of intracellular FXIIIA (as in monocyte-derived macrophages) are not detected in phagocytic or secretory vesicles, indicating that FXIIIA might play a direct role in cell-fate determination (M1 or M2), possibly by altering the cellular substructure [15]. More recently, work published by Alshehri et al. proposed that although monocytes do not contribute to plasma FXIIIA, in the case of VTE, entrapped monocytes expose FXIIIA on their surfaces to the fibrin clot surface [12]. The study reveals differential expression of *F13A1* in the presence of pro-inflammatory and anti-inflammatory cytokine-based stimulation of monocytes. On profiling the array data from M1 and M2 macrophages, strong expression of *F13A1* in M2 was observed; the time-based array indicates that FXIII-A most likely contributes to macrophage polarization (Figure 5). As discussed by Alshehri et al., the monocytes entrapped in thrombus largely have an M2 phenotype, and the fact they expose FXIII is not surprising as it has established roles in wound-healing. The presence of highly correlated genes (linear regression *p* < 0.05) with FXIII in macrophage polarization towards M2-switch supports this notion (See Supplementary Table S1). Additionally, the upstream regulator analyses predicted (by identifying *F13A1* co-expressed genes) that upstream regulators such as growth factors, transcription factors, and interleukins are targeting both *F13A1-* and *F13A1*-correlated genes, which may suggest an association between *F13A1* and macrophage polarization, i.e., macrophage polarization towards an M2 phenotype and increased expression of *F13A1* occur in a temporal manner and are not mutually independent events (Figure 5). There is coexistence of a common upstream regulator covey for *F13A1* and its correlated genes during M0/M1 and M1/M2 switch that conjoins these two events as a cause-and-effect (Figure 5). 

Mice-based knock-out studies using the Cre/lox system indicated that the plasma FXIIIA pool is derived primarily from aortic resident macrophages and not from platelets [14]. The study suggests that not the monocyte-derived macrophages but resident macrophages are responsible for keeping up the plasma pool of FXIIIA, the primary aortic resident macrophages [14]. While we did not find time-point-based resident macrophage data to define the primary transcription factors that contribute to the eventual plasma FXIII levels, we were able to assess the resident macrophages, plaque-derived array data to be analyzed from another perspective. Our analysis from these resident macrophages reveals that the *F13A1* gene acts as a coadjutant in plaque fate, rather than being detrimental towards its progression. The bioinformatics data reveal and support the existence of a strong role of FXIIIA in inflammation, cellular remodeling, and remodeling of atheroma as well. The lack of any significant differential expression in the three stages analyzed here strongly indicates that FXIIIA is directly involved in plaque stability; it plays no role in plaque formation, progression, rupture, and/or healing but in plaque maintenance (Figure 6, also see Supplementary Table S1). The significantly upregulated upstream regulatory element for *F13A1* and its correlated genes is predicted to be “MafB” (z-score 3.945) (Figure 6), which is found to be quintessential for plaque growth by other groups by gene knock out studies [38]. However, the analyzed data set defines that the resident macrophages isolated from the plaque samples are activated. Recent data reported by Verma et al. reveal that *F13A1* overexpression is seen only in the advanced stages of atherosclerotic plaques, and it is likely to be upregulated as a result of cytokine-storm in advanced stages of atherosclerotic plaque [39]. Secondly, our data only take into consideration the resident macrophages derived from plaque.

As a summary and conclusion, analyses and profiling of time-series-based micro-array expression data derived from different cell types expressing (and secreting) FXIIIA and FXIIIB subunit predict the inter-regulation of expression for both the genes. FXIIIA has roles beyond coagulation and is very likely to be involved in pro-inflammatory M2 phenotype switching of monocyte-derived macrophages. Owing to its role as a stabilizer, FXIIIA is likely to be indispensable for plaque maintenance at advanced stages of atherosclerotic plaque; however, is not needed for plaque initiation. Future studies are warranted for establishing effective, direct, and exclusive roles of FXIIIA molecule towards (a) macrophage polarization, (b) regulation of *F13B* gene expression, and (c) progression of early-stage plaque to advanced stages, in thrombus models. 

## Data Availability

The output files of evaluations done for this work are available from the authors upon reasonable request.

## Supplementary Materials

The following supporting information can be downloaded at: https://www.mdpi.com/article/10.3390/ijms23094725/s1.
