# Supplementary material for: Exploring Diverse Coagulation Factor XIII Subunit Expression Datasets: A Bioinformatic Analysis
Source: Int J Mol Sci. 2022 Apr 25;23(9):4725. doi: 10.3390/ijms23094725 (PMC9099568; doi:10.3390/ijms23094725)
Supplement: Supplementary file 1 [file ijms-23-04725-s001.zip › ijms-1684353-supplementary/Supplementary Figure.pdf]

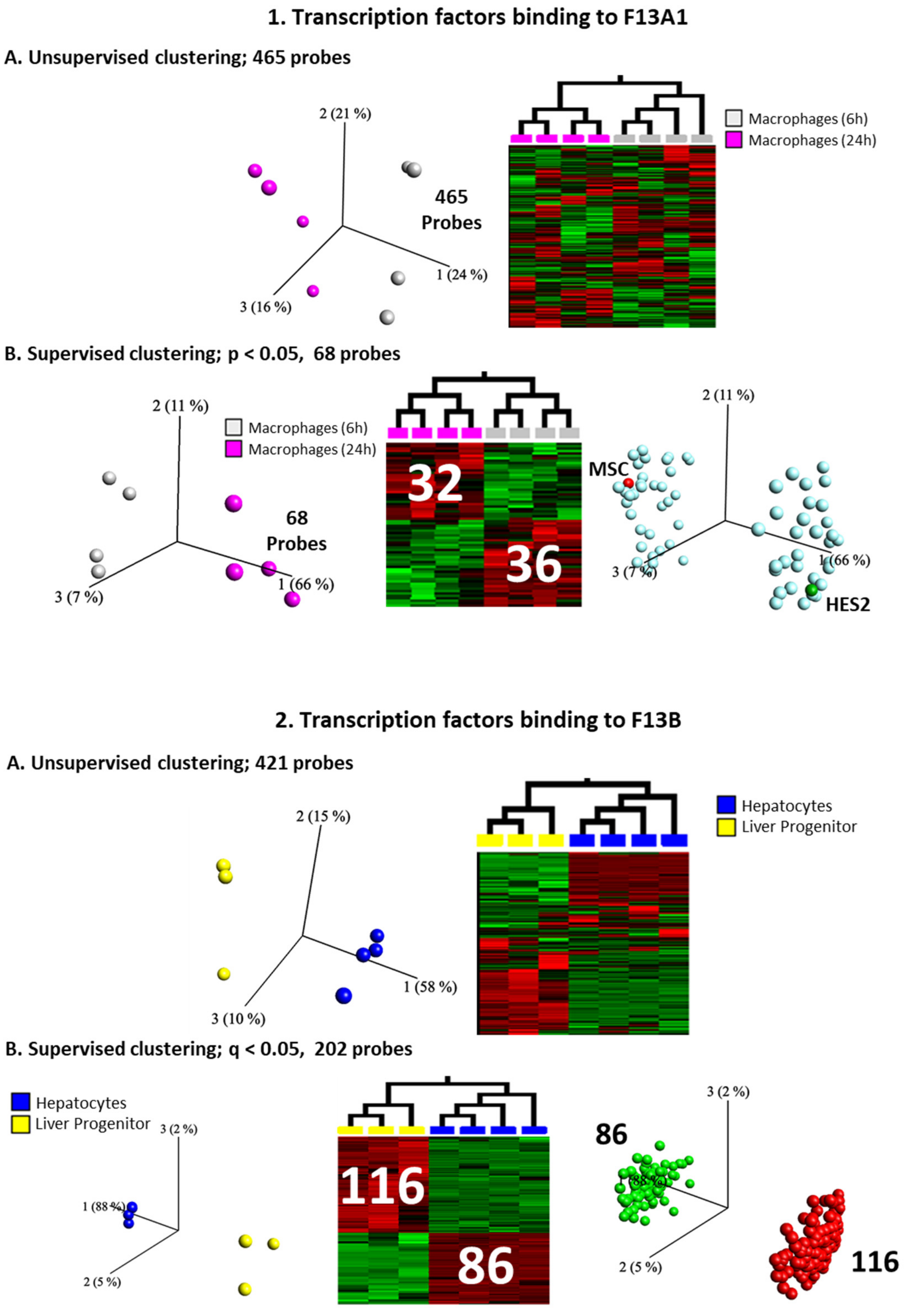

Supplementary Figure S1: Expression analysis of transcription factors binding to 2000bp long-promoter of F13A1 and F13B. 1A) Unsupervised clustering of 465 probes correspond to TFs expressed in 6h and 24hr macrophages. Left: 3D-PCA of 465 probes, Right: Heatmap of 465 probes. 1B) Differentially expressed TFs at $p < 0.05$ between 6h and 24h macrophages. Left: 3D-PCA of 68 differentially expressed TFs, Right: Heatmap of 68 differentially expressed TFs. 2A) Unsupervised clustering of 421 probes correspond to TFs expressed in hepatocytes and liver progenitor cells. Left: 3D-PCA of 421 probes, Right: Heatmap of 421 probes. 1B) Differentially expressed TFs at %of FDR

between hepatocytes and liver progenitor cells. Left: 3D-PCA of 202 differentially expressed TFs, Right: Heatmap of 202 differentially expressed TFs.
